# A Novel Strategy for Minimum Attribute Reduction Based on Rough Set Theory and Fish Swarm Algorithm

**DOI:** 10.1155/2017/6573623

**Published:** 2017-08-15

**Authors:** Yuebin Su, Jin Guo

**Affiliations:** ^1^School of Information Science and Technology, Southwest Jiao Tong University, Chengdu 610031, China; ^2^School of Mathematics and Statistics, Sichuan University of Science & Engineering, Zigong 643000, China

## Abstract

For data mining, reducing the unnecessary redundant attributes which was known as attribute reduction (AR), in particular, reducts with minimal cardinality, is an important preprocessing step. In the paper, by a coding method of combination subset of attributes set, a novel search strategy for minimal attribute reduction based on rough set theory (RST) and fish swarm algorithm (FSA) is proposed. The method identifies the core attributes by discernibility matrix firstly and all the subsets of noncore attribute sets with the same cardinality were encoded into integers as the individuals of FSA. Then, the evolutionary direction of the individual is limited to a certain extent by the coding method. The fitness function of an individual is defined based on the attribute dependency of RST, and FSA was used to find the optimal set of reducts. In each loop, if the maximum attribute dependency and the attribute dependency of condition attribute set are equal, then the algorithm terminates, otherwise adding a single attribute to the next loop. Some well-known datasets from UCI were selected to verify this method. The experimental results show that the proposed method searches the minimal attribute reduction set effectively and it has the excellent global search ability.

## 1. Introduction

Data mining, which was known as knowledge discovery in database, includes extracting knowledge, discovering new patterns, and predicting the future trends from the amounts of data. Nowadays, with an increasing number of applications in different fields, massive volumes of very high-dimensional data were produced; the data mining faces the great challenge. As known to all, much of datasets contain unnecessary redundant attributes, which not only occupy extensive computing resources but also seriously impact the decision-making process. Reducing the unnecessary redundant attributes becomes very necessary for data mining [1]. Attribute reduction (AR) in the rough set theory (RST) removes redundant or insignificant knowledge with keeping the classification ability of the information system the same as before. It was proposed by Pawlak and Sowinski [2]. Now, RST is widely used in many fields such as machine learning, data mining, and knowledge discovery [3–6].

AR is one of the core problems in RST. In particular, minimal reduction problem is an important part of AR in RST, in which the cardinality of attribute subset is the smallest among all possible reductions. It has been paid much attention by many researchers. One basic solution to find the minimal reducts is to construct a discernibility function and simplify it from the dataset by discernibility matrix [7–9]. Unfortunately, it has been shown that the problem of minimal reduct generation is NP-hard and the run time of generating all reducts is exponential [10]. Recently, because many kinds of NP-hard problems can be solved by heuristic algorithms with increasing computational cost, heuristic attribute reduction algorithm is the main research direction in the field of AR [11].

In general, swarm intelligence algorithm is one kind of heuristic approaches which were used widely for solving attribute reduction problem, including genetic algorithm (GA) [12–14], particle swarm optimization (PSO) [15–18], ant colony optimization (CO) [19, 20], and fish swarm algorithm (FSA) [11, 21, 22]. FSA is a kind of evolutionary algorithm which was inspired by the natural schooling behaviors of fish to generate candidate solutions for optimization problems, such as random, swarming, following, and preying behaviors. It has a strong ability to avoid local minimums in order to achieve a global optimization [23]. Due to its abilities to perform, FSA has received much attention in recent years.

In this paper, a new coding method about the subset of attribute sets is proposed. By the coding method, a novel strategy for minimal attribute reduction algorithm based on FSA and RST is proposed. It firstly identifies the core attributes by discernibility matrix. Based on the core attributes, all subsets without containing the core attribute are encoded into an integer by the proposed coding method and an initial population is generated for FSA used to find the optimal set of reducts. The fitness function of a subset is defined based on the attribute dependency of the formed rough set. In each loop, the evolutionary direction of the individual is limited to a certain extent by the coding method. If the maximum attribute dependency and the attribute dependency of condition attribute set are equal, then the algorithm terminates, otherwise, adding a single attribute to the next loop. Different benchmark datasets are used to compare the numerical results; our proposed method is a robust and cheap method for calling the fitness function.

The rest of the paper is organized as follows. In Section 2, we introduced some basic concepts in rough sets and fish swarm algorithm. In Section 3, we focus the coding method of combination set. In Section 4, a novel attribute reduction algorithm based on fish swarm algorithm and rough set is proposed. In Section 5, some well-known datasets are used to test the performance of the proposed method. Finally, Section 6 concludes the paper and the areas of further research.

## 2. Background

### 2.1. Base Notions of Rough Set Theory

In this section, some basic notions and its proposition will be reviewed in the theory of rough set.

A decision table can be represented as *S* = {*U*, *A*, *V*, *f*}, where *U* = {*x*_1_, *x*_2_,…, *x*_*n*_} is a nonempty finite set of objects, *A* = *C* ∪ *D*, where *C* is a set of condition attribute and *D* is a decision attribute set, *V* is the domains of attributes belonging to *A*, and *f* : *U* × *A* → *V* is a function assigning attribute values to objects in *U*.

For any *R*⊆*C* ∪ *D*, there is an associated indiscernibility relation IND(*R*):

(1) INDR=x,y ∣ fx,a=fy,a,  ∀a∈P,  x,y∈U.

Let *X*⊆*U*; the *R*-lower approximation of *X* is defined as $\underline{R}X=\left\{x\in U|{\left[x\right]}_{R}\subseteq X\right\}$ where [*x*]_*R*_ denotes an equivalence class of IND(*R*) determined by object *x*. The notation POS_*R*_(*D*) refers to the *R*-positive region is given by $POS_{R}\left(D\right)={⋃}_{X\in U/IND\left(D\right)}^{}\underline{R}X$. The *R*-approximation quality with respect to decisions attribute set *D* is defined as follows:

(2) $$\begin{array}{c} \gamma_{R}=\frac{\left|POS_{R}\left(D\right)\right|}{\left|U\right|} \end{array}$$

and the core attribute set is defined as

(3) $$\begin{array}{c} Core=\left\{\;a:\gamma_{R\setminus \left\{a\right\}}\neq \gamma_{R}\;\right\}. \end{array}$$

### 2.2. The Principle of FSA

FSA is a new bionic optimization algorithm which simulates the fish swarm behaviors such as preying, swarming, and following behaviors and updates the maximum fitness value on the bulletin board. In FSA, let *N* be the population size, the Artificial Fishes (AF) are generated by random function which is represented by a *D*-dimensional position *X*_*i*_ = (*x*_*i*1_, *x*_*i*2_,…, *x*_*iD*_), and *X*_*i*∣next_ is the updated value of *X*_*i*_. Food satisfaction of *X*_*i*_ is represented as fitness function value *Y*_*i*_ = *F*(*X*_*i*_). The Euclidean distance *d*_*ij*_ = ‖*X*_*i*_ − *X*_*j*_‖ is denoted as the relationship between *X*_*i*_ and *X*_*j*_. Other parameters include step (representing maximum step length), visual (the visual distances of fish), rand being a random number in [0,1], and *σ* (a crowd factor).

Preying behavior is a basic behavior of FSA. As shown in (4), for *X*_*i*_, we randomly select a random *X*_*j*_ within the current visual scope. If *Y*_*i*_ < *Y*_*j*_, then move a step from *X*_*i*_ to *X*_*j*_. Otherwise, *X*_*i*_ move a step to another random *X*_*j*_ that *Y*_*i*_ < *Y*_*j*_. After a number of trials, if the random *X*_*j*_ that meets *Y*_*i*_ < *Y*_*j*_ is not satisfied, *X*_*i*_ will be replaced with a random position within the visual scope directly. It makes the FSA escape from the local optimal solution. Define the prey(*X*_*i*_) function as (4).

Swarming behavior is described as (5). It shows the attraction of the swarm center to the individual. Let *n*_*f*_ be the number of AFs within the current visual scope of *X*_*i*_, and *X*_*i*_^*c*^ is the center position of those neighbors. For the swarm center *X*_*i*_^*c*^, if the food satisfaction is greater and not too crowded (i.e., *σY*_*i*_ < *Y*_*c*_/*n*_*f*_), then move a step from *X*_*i*_ to *X*_*i*_^*c*^. Otherwise, preying behavior is to identify a next position for the current.

Following behavior is described as (6). Let *X*_*i*_^max^ be a AF with the greatest food consistence among AFs in the current visual scope. If the food satisfaction is greater and not too crowded (i.e., *σY*_*i*_ < *Y*_*i*_^max^/*n*_*f*_), then move a step from *X*_*i*_ to *X*_*i*_^max^. Otherwise, preying behavior is to identify a next position for the current.

(4) Xi ∣ next=Xi+rand×step×Xj−XiXj−XiYi<YjXi+rand×stepelse,

(5) Xi ∣ next=Xi+rand×step×Xic−XiXic−XiσYi<YcnfpreyXielse,

(6) Xi ∣ next=Xi+rand×step×Ximax−XiXimax−XiσYi<YmaxnfpreyXielse.

In addition, step and visual parameters play an important role in FSA. They determine the convergence speed of FSA and make it escape from the local optimal solution. They are described as follows [11]:

(7) visual=visual×f4×genGEN,0,2,stepi+1=stepi×ggenGEN,

where *f*( ) is the Lorentzian function and *g*( ) is the normal distribution function.

## 3. A Coding Method for Combination

Let *C* = {1,2,…, *n*} be an integers set which contain *n* elements. The permutations number of *C* is *n*!. Sort them from small to large by lexicographic order. The Cantor expansion and inverse Cantor expansion indicate that there is a one-to-one correspondence between the full permutation set of *C* and {1,2,…, *n*!}. Converting the full permutation into decimal number can be used to solve the TSP problem by the heuristic algorithm. Different from the TSP problem, rough set attribute reduction focuses on the combination of attribute set; it is necessary to discuss the ranking of a combination in the combinations sequence.

Let *C*(*n*, *m*) = {*α*∣*α*⊆*C*, |*α*| = *m*}; then the cardinal number of *C*(*n*, *m*) is *C*_*n*_^*m*^. For *α* ∈ *C*(*n*, *m*) and *α* = (*a*_1_, *a*_2_,…, *a*_*m*_), then *α*⊆*C*. Sort all the elements that, in *α* from small to large, that is,

(8) if  1≤i<j≤m,  then ai<aj.

By lexicographic order, *C*(*n*, *m*) can be regarded as a sequence.

**Example 1 .**

Let *C* = {1,2, 3,4, 5,6, 7} and *m* = 4. All the elements of *C*(7,4) were shown in Table 1.

From Table 1, we can find that the NO 22 is $\left(\begin{array}{cccc} 2 & 3 & 4 & 6 \end{array}\right)$. By lexicographic order, the following proposition is apparent.

**Proposition 2 .**

Let *α* = (*a*_1_, *a*_2_,…, *a*_*m*_), *β* = (*b*_1_, *b*_2_,…, *b*_*m*_) ∈ *C*(*n*, *m*). *α* precede *β* if and only if ∃*k*  (1 ≤ *k* ≤ *m*) such that, ∀*i*  (1 ≤ *i* < *k*), *a*_*i*_ = *b*_*i*_ and *a*_*k*_ < *b*_*k*_.

**Proposition 3 .**

Let *α* = (*a*_1_, *a*_2_,…, *a*_*m*_) ∈ *C*(*n*, *m*), then, ∀*i*  (1 ≤ *i* ≤ *m*),  *i* ≤ *a*_*i*_ ≤ *n* + 1 − *m*.

According to the property of combination, let *α* = (*a*_1_, *a*_2_,…, *a*_*m*_) ∈ *C*(*n*, *m*) and *a*_*i*_ = *j*. Then the number of combinations in *C*(*n*, *m*) as *α* is *C*_*n*−*j*_^*m*−*i*^ by Proposition 3. Thus we can get a matrix *M*(*T*(*i*, *j*))_*m*×*n*_ about *C*(*n*, *m*), where

(9) $$\begin{array}{c} T\left(\;i,j\;\right)=\left\{\begin{array}{cc} C_{n-j}^{m-i} & 1\leq i\leq m,\;\;i\leq j\leq n+1-m\\ 0 & other. \end{array}\right. \end{array}$$

The follow equation is apparent: if *T*(*i*, *j*) ≠ 0, then

(10) $$\begin{array}{c} T\left(\;i,j\;\right)=\overset{n}{\underset{k=j+1}{\sum }}T\left(\;i+1,k\;\right). \end{array}$$

**Example 4 .**

For *C*(7, 4), matrix *M*(*T*(*i*, *j*))_7×4_ is as follows:

(11) $$\begin{array}{c} M=\left(\begin{array}{ccccccc} 20 & 10 & 4 & 1 & 0 & 0 & 0\\ 0 & 10 & 6 & 3 & 1 & 0 & 0\\ 0 & 0 & 4 & 3 & 2 & 1 & 0\\ 0 & 0 & 0 & 1 & 1 & 1 & 1 \end{array}\right). \end{array}$$

Since |{1,2,…, *C*_*n*_^*m*^}| = |*C*(*n*, *m*)| = *C*_*n*_^*m*^, define a mapping *h* from {1,2,…, *C*_*n*_^*m*^} to *C*(*n*, *m*) as follows:

(12) h:1,2,…,Cnm⟶Cn,mhx=α=a1,a2,…,am,

where *x* ∈ {1,2,…, *C*_*n*_^*m*^} and *α* = (*a*_1_, *a*_2_,…, *a*_*m*_) ∈ *C*(*n*, *m*). We will give Algorithm 1 for calculating the combination by the matrix *M*(*T*(*i*, *j*))_*m*×*n*_.

For all *x* ∈ {1,2,…, *C*_*n*_^*m*^}, according to Algorithm 1 and (9) and (10), we can calculate *h*(*x*) = *α* = {*a*_1_, *a*_2_,…, *a*_*m*_}; thus we can decode a integer within *C*_*n*_^*m*^ into a combination in *C*(*n*, *m*).

**Example 5 .**

*C*_7_^4^ = 35; let *x* = 22 ∈ {1,2,…, 35} and *h*(22) = (*a*_1_, *a*_2_, *a*_3_, *a*_4_). By Algorithm 1, we know

 
20 = *T*(1,1) < *x* = 22 < *T*(1,1) + *T*(1,2) = 30; then *a*_1_ = 2, *x* = 22 − *T*(1,1) = 2;
 
*x* = 2 < *T*(2,2 + 1) = 6; then *a*_2_ = 3,  *x* = 2 − 0 = 2;
 
*x* = 2 < *T*(3,3 + 1) = 3; then *a*_3_ = 4,  *x* = 2 − 0 = 2;
 
1 = *T*(4,4 + 1) < *x* = 2 = *T*(4,4 + 1) + *T*(4,4 + 2) = 2; then *a*_4_ = 6;
 
*h*(22) = (2,3, 4,6);
  in Table 1, the 22th permutation being (2,3, 4,6), that is, the same result.

## 4. RFSA-RST Algorithm

In this section, for finding minimal reducts of a dataset, an algorithm which is named RFSA-RST in this paper based on RST and FSA is proposed. It first uses the concepts of the core to find the core attributes, and FSA is employed in a restrained manner to find the minimal reducts to ensure that the result is the minimal length.

### 4.1. Encoding Method

Essentially, AR is a combinatorial optimization problem and the solution space is the power set of the attribute set. Let *C* = {1,2,…, *n*} be conditional attribute set, the power set of *C* be 2^*C*^, and *C*(*n*, *m*) = {*α*∣*α*⊆*C*, |*α*| = *m*}; then *C*(*n*, *m*)⊆2^*C*^. Clearly, ∑_*i*=0_^*n*^*C*(*n*, *i*) = *C* and *C*(*n*, *i*)∩*C*(*n*, *j*) = *ϕ*  (*i* ≠ *j*). By the cardinality of the subset of *C*, 2^*C*^ is split into *n* disjoint parts. According to Algorithm 1, every integer within *C*_*n*_^*m*^ can be decoded into a combination in *C*(*n*, *m*), so integer encoding method will be adopted. Thus, each integer represents a combination in *C*(*n*, *m*) and the solution space is integer type. Let Δ be defined as the increment of *X*_*i*_. By (4)–(6), it may be decimal. So, it is not adaptive to the solution space. In order to improve this problem, the new *X*_*i*_ increment is shown as follows [11]:

(13) $$\begin{array}{c} X_{i\;|\;next}=\left\{\begin{array}{cc} X_{i}+round\left(\Delta \right)+1 & \Delta >0\\ X_{i}+round\left(\Delta \right)-1 & else. \end{array}\right. \end{array}$$

### 4.2. Fitness Function

In FSA, the quality of a AF is determined by fitness function. For AR problem, the subset should retain the classification ability and has minimal attributes. Generally, the fitness function must meet those issues. In addition, it should be as simple as possible to improve the computation efficiency. In this paper, all subsets without containing the core attribute are encoded into an integer as a AF. Since the cardinality of each combination in *C*(*n*, *m*) is fixed, FSA is restricted to a fixed length subset of condition attribute set and judges a AF by its dependency value for the attribute subset represented by the integer, as explained below.

For a AF and its position being *X*, calculate the corresponding combination *h*(*x*) in *C*(*n*, *m*) by Algorithm 1. Define the fitness value of *X* as follows:

(14) $$\begin{array}{c} F\left(\;X\;\right)=\gamma_{h\left(X\right)\cup Core}. \end{array}$$

### 4.3. Algorithm Description

In FSA, the initial population of size *N* needs to be provided representing multiple points in the search space. If there is a fitness value of AF that is equal to *γ*_*C*_, then the process terminates and outputs the corresponding attribute subsets of AF. If change in average fitness value of two successive generations is 0.001 or less, no further generations are created and terminate this loop. Then a new initial population is created for the next loop. These attribute subsets of the new initial population correspond to one more attribute. So, initial strings in *i*th loop of the algorithm have one more attribute than that in (*i* − 1)th loop. The whole process is repeated based on this new initial population until a minimal set of reduct(s) is found. The detailed process of RFSA-RST is shown in Algorithm 2.

## 5. Performance Comparison

To evaluate the effectiveness of the proposed algorithm, RFSA-RST, we carried out experiments in the following way. Another two different types of algorithms were used. One is proposed in [12], denoted here by RGA-RST, and the other is proposed in [17], denoted here by PSO-RST. A PC running Windows XP with 3.1 GHz CPU and 1 GB of main memory was employed to run these three algorithms. The test datasets were obtained from the UCI machine learning repository and 6 datasets were chosen. Basic information about the datasets is listed in Table 2, where *n* and *m* are the number of objects and (condition) attributes, respectively.

To make the comparison fair and reasonable, the three algorithms were independently run 50 times on each of the datasets with the same setting of the parameters involved. For each run, three values needed to be recorded for each experiment, the length of the output, the run time, and whether the output is a reduction. If the result is a reduction, then the run is said to be normal; otherwise, the run is said to be unsuccessful. If the result corresponds to a minimum reduct, then the run is not only normal but also successful. Let STL, AVL, and AVT be denoted as the shortest length, average length, and average running time, respectively, during the 50 runs. The ratios of successful and normal runs are denoted, respectively, by *s*_1_ and *s*_2_. The performances of the three algorithms on the datasets are reported in Tables 3 and 4, respectively.

From Table 3, the proposed algorithm has the same performance as the other two algorithms for the shortest length of the outputs, but outperforms the other two algorithms in terms of the average length of output solutions except for the Soybean-small dataset. It means that the stability of the proposed algorithm is higher than the other two algorithms.

From Table 4, for the ratio of normal runs, all the outputs of the three algorithms are roughly the same, but the proposed algorithm outperforms the other two algorithms in terms of the ratio of successful runs except for the Soybean-small dataset. Therefore, if the minimum attribute reduction is required, the proposed algorithm is the best of the three algorithms. It is also reflected to the average length in Table 3. As far as the average running time is concerned, the proposed algorithm is slightly worse than PSO-RST but better than RGA.

**Figure 1:**
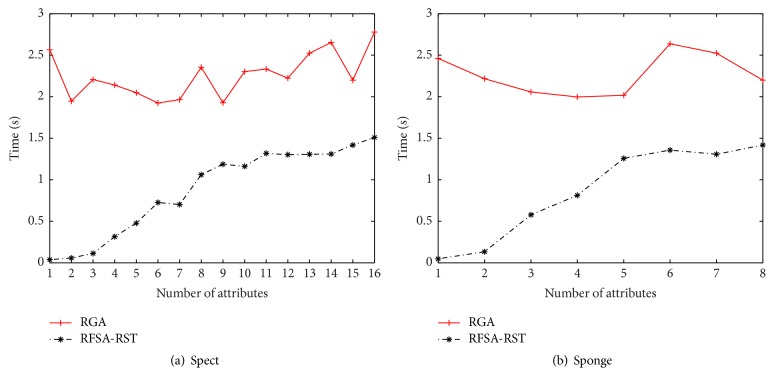
Average running time.

Since the operation modes of the proposed algorithm and RGA algorithm are the same, another experiment has been done to evaluate the time efficiency of the two algorithms. We modify some process of both algorithms where the core attribute set is no longer identified and *n* in RGA (for the parameter *m* in the proposed algorithm) is set from 1 to the cardinality of minimal attribute reduction for each dataset. Record the running time for each loop. In order to show the results more clearly and length limitations, we only show the test results about the datasets Spect and Sponge. As shown in Figure 1. By Figure 1, we can find that, for all datasets, the running time in each loop of the proposed algorithm was increased with the increase of *m*, while RGA were relatively stable. That is due to the fact that the search process of the proposed algorithm focuses on the attribute subset with fixed length. Therefore, the time efficiency of the algorithm is higher in each loop and the convergence rate is faster. It shows that the algorithm proposed in this paper is more efficient.

Summing up the experiment results, we see that the proposed algorithm is more efficient than the other two typical algorithms on the datasets for test, although the running time is slightly worse than PSO-RST.

## 6. Conclusions

In this paper, we derived a new method for minimum attribute reduction based on rough set theory and fish swarm algorithm. In this method, by a coding method of combination subset of attributes set, the FSA has been used to search minimum attribute reduction and attribute dependency has been applied to calculate the fitness values of the attribute subsets. The FSA has been restrained in which every integer corresponds to a specified length attribute subset in each search process. The cardinality of the attribute subset represented by AF was starting from the length of the core and incremented by one after in each loop.

Numerous test results show that it can improve not only the accuracy of minimal attribute reduction but also the efficiency of convergence rate.

To improve search efficiency, future enhancements to this work are to confirm whether the starting length was reasonable and, to improve the search time efficiency, these works can reduce redundant search process.

## Figures and Tables

**Algorithm 1:**
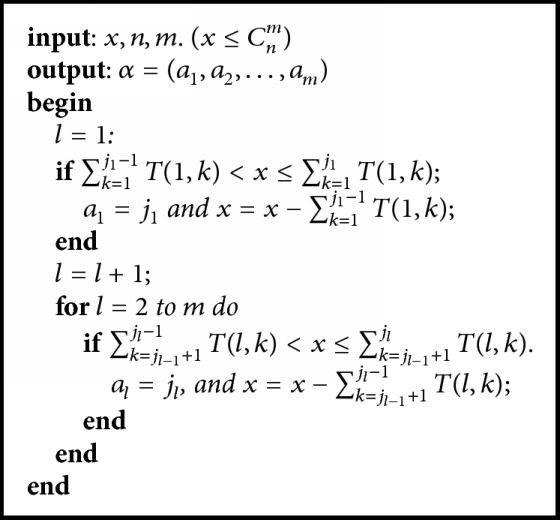
DetoCo(*x*, *n*, *m*).

**Algorithm 2:**
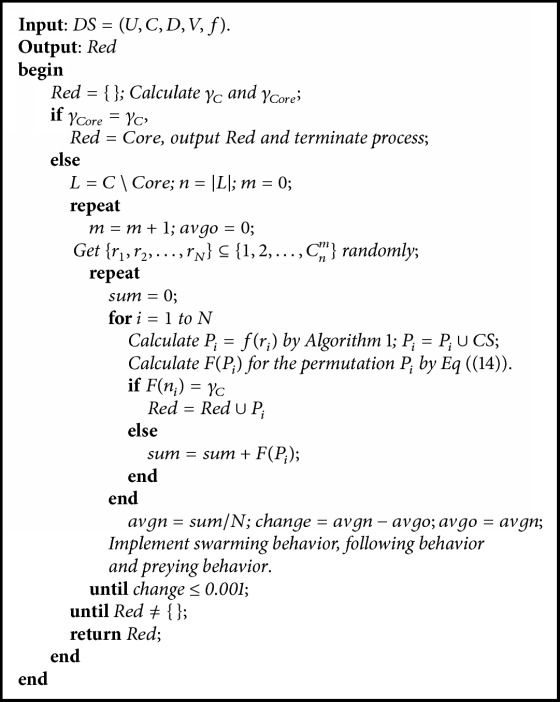
RFSA-RST(DS).

**Table 1:** *C*(7,4).

1	$\left(\begin{array}{cccc} 1 & 2 & 3 & 4 \end{array}\right)$	8	$\left(\begin{array}{cccc} 1 & 2 & 5 & 6 \end{array}\right)$	15	$\left(\begin{array}{cccc} 1 & 3 & 5 & 7 \end{array}\right)$	22	$\left(\begin{array}{cccc} 2 & 3 & 4 & 6 \end{array}\right)$	29	$\left(\begin{array}{cccc} 2 & 4 & 6 & 7 \end{array}\right)$
2	$\left(\begin{array}{cccc} 1 & 2 & 3 & 5 \end{array}\right)$	9	$\left(\begin{array}{cccc} 1 & 2 & 5 & 7 \end{array}\right)$	16	$\left(\begin{array}{cccc} 1 & 3 & 6 & 7 \end{array}\right)$	23	$\left(\begin{array}{cccc} 2 & 3 & 4 & 7 \end{array}\right)$	30	$\left(\begin{array}{cccc} 2 & 5 & 6 & 7 \end{array}\right)$
3	$\left(\begin{array}{cccc} 1 & 2 & 3 & 6 \end{array}\right)$	10	$\left(\begin{array}{cccc} 1 & 2 & 6 & 7 \end{array}\right)$	17	$\left(\begin{array}{cccc} 1 & 4 & 5 & 6 \end{array}\right)$	24	$\left(\begin{array}{cccc} 2 & 3 & 5 & 6 \end{array}\right)$	31	$\left(\begin{array}{cccc} 3 & 4 & 5 & 6 \end{array}\right)$
4	$\left(\begin{array}{cccc} 1 & 2 & 3 & 7 \end{array}\right)$	11	$\left(\begin{array}{cccc} 1 & 3 & 4 & 5 \end{array}\right)$	18	$\left(\begin{array}{cccc} 1 & 4 & 5 & 7 \end{array}\right)$	25	$\left(\begin{array}{cccc} 2 & 3 & 5 & 7 \end{array}\right)$	32	$\left(\begin{array}{cccc} 3 & 4 & 5 & 7 \end{array}\right)$
5	$\left(\begin{array}{cccc} 1 & 2 & 4 & 5 \end{array}\right)$	12	$\left(\begin{array}{cccc} 1 & 3 & 4 & 6 \end{array}\right)$	19	$\left(\begin{array}{cccc} 1 & 4 & 6 & 7 \end{array}\right)$	26	$\left(\begin{array}{cccc} 2 & 3 & 6 & 7 \end{array}\right)$	33	$\left(\begin{array}{cccc} 3 & 4 & 6 & 7 \end{array}\right)$
6	$\left(\begin{array}{cccc} 1 & 2 & 4 & 6 \end{array}\right)$	13	$\left(\begin{array}{cccc} 1 & 3 & 4 & 7 \end{array}\right)$	20	$\left(\begin{array}{cccc} 1 & 5 & 6 & 7 \end{array}\right)$	27	$\left(\begin{array}{cccc} 2 & 4 & 5 & 6 \end{array}\right)$	34	$\left(\begin{array}{cccc} 3 & 5 & 6 & 7 \end{array}\right)$
7	$\left(\begin{array}{cccc} 1 & 2 & 4 & 7 \end{array}\right)$	14	$\left(\begin{array}{cccc} 1 & 3 & 5 & 6 \end{array}\right)$	21	$\left(\begin{array}{cccc} 2 & 3 & 4 & 5 \end{array}\right)$	28	$\left(\begin{array}{cccc} 2 & 4 & 5 & 7 \end{array}\right)$	35	$\left(\begin{array}{cccc} 4 & 5 & 6 & 7 \end{array}\right)$

**Table 2:** Basic information about the datasets.

Data set	*n*	*m*
Lung-cancer	26	52
Soybean-small	47	36
Sponge	76	45
Zoo	101	17
Blance	625	5
Spect	187	23

**Table 3:** Performance of three algorithms.

Dataset	PSO-RST		RGA		RFSA-RST	
	STL	AVL	STL	AVL	STL	AVL
Lung-cancer	3	4.14	3	4.02	3	**3.92**
Soybean-small	2	2.29	2	**2.32**	2	2.38
Sponge	8	8.7	8	8.4	8	**8.02**
Zoo	5	5.32	5	5.05	5	**5**
Blance	4	4.02	4	4	4	4
Spect	16	16	16	16	16	16

**Table 4:** Performance of three algorithms.

Dataset	PSO-RST		RGA		RFSA-RST	
	*s*_1_/*s*_2_	AVT(s)	*s*_1_/*s*_2_	AVT(s)	*s*_1_/*s*_2_	AVT(s)
Lung-cancer	0.06/1	0.2073	0.08/1	0.8103	**0.22**/1	0.2255
Soybean-small	0.7/1	0.7117	**0.78**/1	1.0177	0.72/1	0.7163
Sponge	0.6/0.92	6.4159	0.62/0.98	16.1129	**0.78**/0.98	6.8089
Zoo	0.94/1	1.3892	0.96/1	2.1648	**1**/1	1.4584
Blance	0.98/1	0.5726	1/1	1.9935	1/1	0.6045
Spect	1/1	12.0372	1/1	36.0937	1/1	12.9012
